# Un-resolving frozen shoulder: Are we really treating it?

**DOI:** 10.12669/pjms.40.1.7440

**Published:** 2024

**Authors:** Abrar Ahmed Wagan, Paras Surahyo

**Affiliations:** 1 Abrar Ahmed Wagan, MBBS, FCPS (Medicine), FCPS (Rheumatology), FACR. Associate Professor of Rheumatology, Indus Medical College, Tando Mohammad Khan, Pakistan; 2 Paras Surahyo, MBBS, FCPS (Radiology) Assistant Professor, Department of Radiology, Bilawal Medical College, Jamshoro, Pakistan

**Keywords:** Shoulder pain, Rotator cuff tendinopathy, Adhesive capsulitis, Ultrasound

## Abstract

**Objective:**

To perform ultrasound examination in un-resolving frozen shoulder disorder, in Pakistani cohort visiting rheumatology clinic.

**Methods:**

This cross sectional study was carried out at Department of Rheumatology, Indus Medical College Tando Mohhamad Khan, from 16^th^ March 2022 to 30^th^ October 2022. Patients diagnosed as unilateral frozen shoulder on clinical grounds and received intra-articular injection (s) in last six months, never been investigated, still persisting with pain and restricted range of shoulder motion were enrolled. After the demographic details and shoulder examination, ultrasound examination of both shoulder joints was performed by senior musculoskeletal radiologist, to know the exact diagnosis.

**Results:**

In 138 cases on ultrasound examination following injuries were noted: rotator cuff tendinopathy (RCT) (61%), adhesive capsulitis (21%), mixed lesion (rotator cuff tendinopathy and adhesive capsulitis) (14%).In age group < 50 years rotator cuff tendinopathy was the major lesion, while in cases >50 years age group: adhesive capsulitis (AC) was predominant lesion (p-0.05).Rotator cuff tendinopathy had significant association with supraspinatus tears (p<0.5).

**Conclusion:**

In Un-resolving frozen shoulder pain, ultrasound examination of involved joint helps in reaching the exact cause which may differ from the existing diagnosis and guides to further management.

## INTRODUCTION

In western world shoulder pain is one of the most common musculoskeletal problems, with a one-year prevalence of 47% and lifetime prevalence of up to (70%).Rotator cuff tendinopathy, an umbrella term, affecting subacromial structures like rotator cuff tendinitis/ tendinosis, subacromial bursitis and shoulder impingement syndrome, characterized by pain and weakness during external rotation and elevation, leading to impaired activities of daily living with significant socio-economic impact, due to loss of work and treatment costs and it is refractory to treatments (oral NSAID’s, physiotherapy, and intraarticular steroid injections).^1^-^3^ Adhesive capsulitis also known as (frozen shoulder), is a common pathological condition that causes pain and significantly restricts the active and passive range of motion, diagnosed on the basis of clinical suspicion, while imaging only being used to exclude other causes.

It is of uncertain aetiopathogenesis, in absence of intrinsic shoulder disease, causes can be divided into primary (idiopathic) and secondary, with the latter developing after stiffness and immobility caused by a previous shoulder trauma or surgery, calcific tendinopathy of the rotator cuff tendons during the painful resorptive phase, or a systemic dysmetabolic, endocrine, rheumatological, immunological, neurological, cardiac disorder, malignancy, and sometimes dupuytren’s disease.^4^,^5^ The prevalence of adhesive capsulitis in general population is about 2-5% but in diabetic patients it is (> 20%), while (70%) sufferers are women, diabetics have worse functional outcomes than normal and in both genders its refractory to treatment.^6^,^7^

Rotator cuff tendinopathy is the most common cause of shoulder pain in adults, with a prevalence of (>20%) and diabetics had a (2.11-fold) higher risk of tendiopathy, as high blood glucose level, affects tendons collagen crosslinking, for its diagnosis radiographs are the standard initial imaging study these may suggest problem, but unable to quantify tears, so the most sensitive first-line imaging modality for early rotator cuff lesion includes, ultrasound (US), MRI, and direct MR arthrography (MRA).^8^,^9^

In Shoulder pain disorders, shoulder impingement syndrome, earliest feature of rotator cuff injury has highest prevalence (36%).^10^ Rotator cuff injury can itself causes adhesive capsulitis, because of pain and disuse and no test (laboratory or imaging) alone provides the definitive diagnosis of the adhesive capsulitis.^11^ Common and mixed shoulder disorders presents with similar clinical features, and poor consensus on diagnostic criteria and concordance in clinical assessment leads to complication in treatment options.^12^

Clinicians usually makes mistakes by not diagnosing impingement syndrome rather frozen shoulder by visual diagnosis or sometime they both exists but treated as one entity and most of time cases are not investigated and treated properly leading to increased burden on patient and health care system. Ultrasound examination plays a vital role in real time diagnosis in such cases.

## METHODS

After IRB (IRB32/2022) approval this cross-sectional study was conducted at Department of Rheumatology Indus Medical College Tando Mohammad Khan from 16-03-2022 to 30-10-2022. Total 138 patients were selected with history of unilateral shoulder pain >6 months, diagnosed as adhesive capsulitis/frozen shoulder received treatment for it in the form intra-articular corticosteroid injections (single or multiple times) and never had imaging study, provided informed consent, patients with history of road traffic accident, surgery/manipulation for neck or shoulder joint, stroke, cervical radiculopathy, metastatic bone disease, autoimmune diseases (RA, SLE, mixed connective tissue disorder), Parkinson disease, thyroid disorders were excluded.

After the demographic details history and clinical examination of shoulder joint was carried out by consultant Rheumatologist for painful arc, tenderness, muscle atrophy and other joints examination if required than sent to radiologist having more than five years’ experience in Musculoskeletal sonography. Clinical information was kept hidden. Radiologist performed ultrasound of both shoulder joints with (linear probe 6-12 MHz) for following features of rotator cuff tendinopathy: Subacute impingemnt, supraspinatus tear, infraspinatus tear, subacromion bursitis-sub-deltoid bursitis, gleno-humeral effusion, calcific tendinitis, for the adhesive capsulitis: examined the axillary pouch (AP), coracohumeral ligament(CHL), superior glenohumeral ligament (SGHL), the long head of the biceps tendon, and dynamic evaluation of shoulder range of motion. The maximum thicknesses of the CHL and SGHL were compared with those in the unaffected contralateral shoulder, and axial measurements were made at the level of the pulley with the LHBT in short-axis view.

Data were stored and analyzed using IBM-SPSS version 23.0.Frequencies with percentages were reported on age group, gender, disease duration, rotator cuff tendinopathy, adhesive capsulitis, mixed lesion and other studied variables. Pearson chi square test was used to check the association of sub acromion impingement, adhesive capsulitis, and mixed lesion with gender, age group and sub acromion impingement with muscle tears. P-values less than 0.05 were considered statistically significant. Bar charts were also used to give graphical presentation of findings.

## RESULTS

There were 138 patients with mean age of 46.7 (SD=±10.1) years, males (56.5%), disease duration of >12 months in (46.4%) cases, (Table-I). Over all frequency of different lesions rotator cuff tendinopathy (61.6%), adhesive capsulitis (24.6%) and mixed lesions (16.7%). (Bar chart.1)

**Table-I:** Baseline Characteristics of the participants (n=138)

Characteristics		Number	(%)
Age Group	<40 years	34	24.6
	40-50 years	66	47.8
	>50 years	38	27.5
	Mean (±SD)	46.7	±10.1
Gender	Female	60	43.5
	Male	78	56.5
Disease Duration	<6 Months	37	26.8
	6-12 Months	37	26.8
	>12 Months	64	46.4

**Figure:**
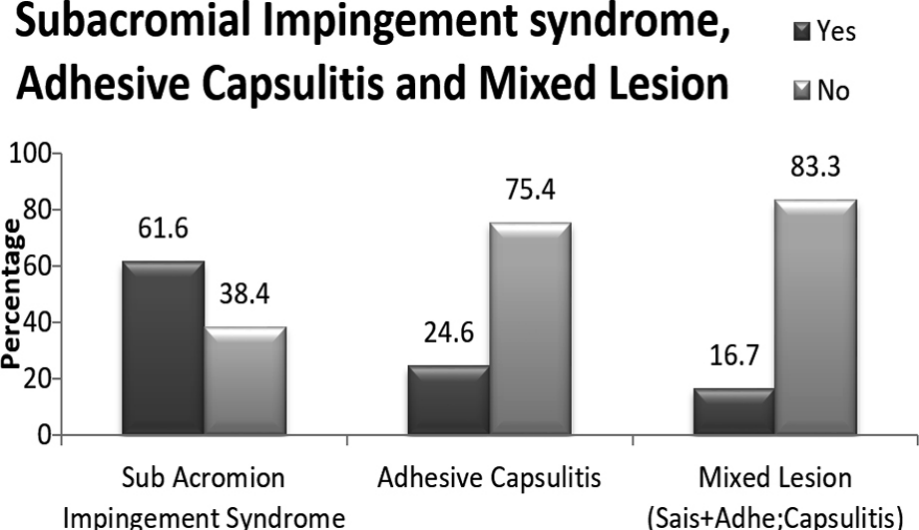
Bar Diagram.^1^

On gender distribution in females rotator cuff tendinopathy (70%), adhesive capsulitis (13.3%) and mixed lesions (20%).In males rotator cuff tendinopathy (55.1%),adhesive capsulitis (33.3%) and mixed lesions (14.1%) p<0.05, (Table-II).

**Table-II:** Association of subacromial Impingement syndrome, Adhesive Capsulitis and Mixed Lesion with Gender.

Variables		Gender				p-value

		Female (n=60)		Male (n=78)		

		n	%	n	%	
Rotator cuff tendinopathy	Yes	42	70.0	43	55.1	0.07
	No	18	30.0	35	44.9	
Adhesive capsulitis	Yes	8	13.3	26	33.3	<0.01*
	No	52	86.7	52	66.7	
Mixed lesion (SIS+AC)	Yes	12	20.0	11	14.1	0.35
	No	48	80.0	67	85.9	

* p<0.05 was considered statistically significant using Pearson Chi Square test.

In age <40-years: Rotator cuff tendinopathy (91.2%), adhesive capsulitis (5.9%), mixed lesions (2.9%), 40-50 years: rotator cuff tendinopathy (62.1%), adhesive capsulitis (21.1%) mixed lesion (16.7%), age >50 years: adhesive capsulitis (47.4%),rotator cuff tendinopathy (34.2%),mixed lesions (28.9%) p<0.05. (Table-III)

**Table-III:** Association of Rotator cuff tendinopathy, Adhesive Capsulitis and Mixed Lesion with Age.

Variable		Age Group						p-value

		<40 years (n=34)		40 - 50 years (n=66)		>50 years (n=38)		

		n	%	n	%	n	%	
Rotator cuff tendinopathy	Yes	31	91.2	41	62.1	13	34.2	<0.01*
	No	3	8.8	25	37.9	25	65.8	
Adhesive capsulitis	Yes	2	5.9	14	21.2	18	47.4	<0.01*
	No	32	94.1	52	78.8	20	52.6	
Mixed lesions	Yes	1	2.9	11	16.7	11	28.9	0.01*
	No	33	97.1	55	83.3	27	71.1	

* p<0.05 was considered statistically significant using Pearson Chi Square test.

Frequency of supraspinatus tear (60%) and infraspinatus tear (21.2%) were present (p<0.05), (Table-IV). In our study interestingly we found that there were mixed lesions (rotator cuff lesions and adhesive capsulitis) existing simultaneously.

**Table-IV:** Rotator cuff tendinopathy with Muscle tears.

Tear		Rotator cuff tendinopathy				p-value

		Yes (n=85)		No (n=53)		

		n	%	n	%	
Supraspinatus tear (complete)	Yes	51	60	15	28.3	<0.01*
	No	34	40	38	71.7	
Infraspinatus tear (complete)	Yes	18	21.2	9	17	0.54
	No	67	78.8	44	83	

* p<0.05 was considered statistically significant using Pearson Chi Square test.

## DISCUSSION

In chronic shoulder pain it is very difficult to know the exact cause of problem, although MRI is study of choice for such cases but due to its un-availability and cost it becomes difficult to get it. In our study we found that those patients who were treated as adhesive capsulitis clinically were in fact suffering from rotator cuff tendinopathy (61%) adhesive capsulitis (24.6%) on ultrasound examination.

Sonography, has a high sensitivity and specificity (100 and 87%) for diagnosis of adhesive capsulitis.^13^ Ultrasound has pooled (sensitivity 79% and specificity 94%) in diagnosing shoulder impingement syndrome with high accuracy in diagnosing the full-thickness tear of the rotator cuff then partial-thickness tear.^14^ Subacromion impingement syndrome is frequently accompanied by a partial or total tear of the rotator cuff tendon, necessitating examination of rotator cuff integrity in these individuals .^15^ In current cohort complete supraspinatus tear was in (n=51) and complete infraspinatus tear (n=18) with rotator cuff lesions (n=85).An Egyptian study taking MRI as gold standard for shoulder problems found that MRI detected rotator cuff tendinopathy (44.4%), partial tears (27.8%) full thickness tears (27.8% ) ultrasound diagnosed, rotator cuff tendinopathy (41.7%),partial tear (22.2%) and full thickness tears (25%).^16^

Contrast enhanced US-arthrography is a new form of shoulder arthrography by using microbubble contrast agents. Intra-articular filling defects, synovitis-like abnormality in the joint are characteristic US-arthrography findings, which are more sensitive than US for diagnosing adhesive capsulitis.^17^

The prevalence of rotator cuff tendinitis rises with age, affected patients are generally over 40 and up to 30% over age 70 have a total defect, but 75% of such cases are asymptomatic.^18^ Angela Cadogan in her study found that eighty patients with frozen shoulder were referred for Orthopaedic evaluation, from general practice, only fifteen patients (19%) were having frozen shoulder.^19^

In current study cases with <50 years age group: rotator cuff lesion was more common with frequency of (62.1%), adhesive capsulitis (21.1%) and >50 years age, adhesive capsulitis (47.1%), rotator cuff tendinitis (34%) (p<0.05).

Khosravi F et al. reported that the point and lifetime prevalence of shoulder pain in middle-aged Iranian women were 18.6% and 27.6%, significant association between present shoulder pain and history of shoulder pain, trauma, osteoporosis, trapezius muscle pain, and cervical radiculopathy (p < 0.05), no significant association between present shoulder pain and diabetes mellitus or postural deviation.^20^

Major risk factors for rotator cuff tendinopathy are; hypercholesterolemia, relative with same problem, excessive lifting, above-shoulder work, hand-held vibration tools usages, age older than 60 years, while adhesive capsulitis has association with diabetes and thyroid disorder.^21^ Rotator cuff tendinitis puts a huge burden on the healthcare system with (28.8%) of people seeking general practitioner consultation, causing high levels of disability and associated healthcare costs.^22^ Shwan Khoschnau in 106 patients found 64 (60%) had shoulder problems, and prevalence of full-thickness cuff tears was 30% (21% of all the 212 shoulders), and (61%) of them had symptoms compared to 33% of the shoulders without tears.^24^ In a Chinese study, frequency of adhesive capsulitis was (11.8%), mostly affected were from middle to elderly age groups.^25^

In contrast this study results shows cases <50 years had rotator cuff lesions and those >50 years had adhesive capsulitis (24.6%).

In a systematic review to assess the prevalence of shoulder pain in occupational groups, stratified by age, results shows its increasingly common over the age of 50 years, in the workforce of physically demanding jobs, but results were conflicting regarding sedentary jobs.^24^ Around 3% of adults in the UK will consult their general practitioner for shoulder pain, with (50%) continue to have pain upto12 months after first consultion.^26^

In some cases rotator cuff tendinitis (calcific tendinitis) starts as adhesive capsulitis as non-solid calcifications penetrates the articular surface and induce an acute reactive inflammatory response (chemical arthritis).^27^ A local study has identified risk factors like long working hours, stress, inappropriate posture, sedentary lifestyle, stoop sitting while working on a computer, reading a book, or in any occupation which involves stoop posture.^28^

### Limitations

This study has certain shortcomings like; sample size is small, it’s a cross sectional study so results can’t be generalized, but to best of our knowledge this is first study which has been carried out in un-resolving shoulder joint pain, with ultrasound examination to know the exact cause of persistent shoulder joint pain in local population.

## CONCLUSION

Shoulder joint pain is very common, its proper diagnosis and effective treatment is warranted in every patient. Ultrasound examination by a trained musculoskeletal radiologist plays a vital role in making the real time diagnosis, it is available everywhere, cost effective, easy to perform, so for its proper utilization awareness and training is required to rheumatologist, orthopedics and radiologists.

### Authors’ Contribution:

**AAW:** Design, drafting, data acquisition data analysis, data interpretation, accuracy, final approval and Responsible for the study.

**PS:** Data acquisition, data analysis, data interpretation, drafting, final approval, Accuracy.
